# Machine Learning for Predicting Postoperative Functional Disability and Mortality Among Older Patients With Cancer: Retrospective Cohort Study

**DOI:** 10.2196/65898

**Published:** 2025-05-14

**Authors:** Yuki Hashimoto, Norihiko Inoue, Takuaki Tani, Shinobu Imai

**Affiliations:** 1 Department of Clinical Data Management and Research, Clinical Research Center, National Hospital Organization Headquarters, 2-5-21 Higashigaoka, Meguroku, 152-8621, Japan, 81 3-5712-5133, 81 3-5712-5088; 2 Department of Pharmacoepidemiology, Showa University Graduate School of Pharmacy, Shinagawaku, Japan

**Keywords:** older patients with cancer, postoperative outcomes, functional disability, machine learning, decision-making

## Abstract

**Background:**

The global cancer burden is rapidly increasing, with 20 million new cases estimated in 2022. The world population aged ≥65 years is also increasing, projected to reach 15.9% by 2050, making cancer control for older patients urgent. Surgical resection is important for cancer treatment; however, predicting postoperative disability and mortality in older patients is crucial for surgical decision-making, considering the quality of life and care burden. Currently, no model directly predicts postoperative functional disability in this population.

**Objective:**

We aimed to develop and validate machine-learning models to predict postoperative functional disability (≥5-point decrease in the Barthel Index) or in-hospital death in patients with cancer aged ≥ 65 years.

**Methods:**

This retrospective cohort study included patients aged ≥65 years who underwent surgery for major cancers (lung, stomach, colorectal, liver, pancreatic, breast, or prostate cancer) between April 2016 and March 2023 in 70 Japanese hospitals across 6 regional groups. One group was randomly selected for external validation, while the remaining 5 groups were randomly divided into training (70%) and internal validation (30%) sets. Predictor variables were selected from 37 routinely available preoperative factors through electronic medical records (age, sex, income, comorbidities, laboratory values, and vital signs) using crude odds ratios (*P*<.1) and the least absolute shrinkage and selection operator method. We developed 6 machine-learning models, including category boosting (CatBoost), extreme gradient boosting (XGBoost), logistic regression, neural networks, random forest, and support vector machine. Model predictive performance was evaluated using the area under the receiver operating characteristic curve (AUC) with 95% CI. We used the Shapley additive explanations (SHAP) method to evaluate contribution to the predictive performance for each predictor variable.

**Results:**

This study included 33,355 patients in the training, 14,294 in the internal validation, and 6711 in the external validation sets. In the training set, 1406/33,355 (4.2%) patients experienced worse discharge. A total of 24 predictor variables were selected for the final models. CatBoost and XGBoost achieved the largest AUCs among the 6 models: 0.81 (95% CI 0.80-0.82) and 0.81 (95% CI 0.80-0.82), respectively. In the top 15 influential factors based on the mean absolute SHAP value, both models shared the same 14 factors such as dementia, age ≥85 years, and gastrointestinal cancer. The CatBoost model showed the largest AUCs in both internal (0.77, 95% CI 0.75-0.79) and external validation (0.72, 95% CI 0.68-0.75).

**Conclusions:**

The CatBoost model demonstrated good performance in predicting postoperative outcomes for older patients with cancer using routinely available preoperative factors. The robustness of these findings was supported by the identical top influential factors between the CatBoost and XGBoost models. This model could support surgical decision-making while considering postoperative quality of life and care burden, with potential for implementation through electronic health records.

## Introduction

The global cancer burden is rapidly increasing, with an estimated 20 million new cases and 9.7 million deaths in 2022 [1]. In Japan, the lifetime risk of being diagnosed with cancer is approximately 65.5% and 51.2% for men and women, respectively [2].

In addition, the global population is aging rapidly, with the proportion of those aged ≥65 years expected to increase from 9.1% in 2019 to 15.9% by 2050 [3]. Japan faces the most advanced stage of this demographic shift, with the population of older adults expected to increase from 28.8% in 2020 to 37.7% by 2050 [4]. Thus, cancer control for older patients has become an urgent issue worldwide, including in Japan.

Older patients with cancer often face challenges such as frailty [5], comorbidities [6-8], and socioeconomic status [6], which affect treatment outcomes and quality of life (QOL). Surgical resection is a key treatment, which requires careful consideration in older patients with cancer due to concerns about postoperative functional disability and its impact on long-term outcomes [9]. Some factors may influence surgical outcomes in older patients, including age [9], anemia [10], BMI [11,12], dementia [6,7], frailty [5], low household income [6], malnutrition [13], and smoking [14].

Considering the postoperative QOL and care burden on patients’ families and society, it is important to predict not only postoperative mortality but also functional disability [15]. Hospital-associated disability, defined as functional disability following acute hospitalization, is recognized as a crucial outcome in older patients with significant impact on health care costs and long-term prognosis [16,17]. Some models can predict postoperative mortality [5,13]; however, few have addressed functional disability. A model after lower-extremity surgery [6] could predict the risk of in-hospital mortality and discharge to a nursing home, which is a surrogate for functional disability, in patients admitted from home. Currently, no model can directly predict functional disability after cancer surgery.

Therefore, this study aimed to develop a machine learning–based model for predicting postoperative functional disability and in-hospital mortality in patients with cancer aged ≥65 years using data from 70 hospitals across Japan. This approach will enable patients and their families to make informed decisions about undergoing cancer surgery, considering their postoperative QOL and care burden.

## Methods

### Study Design and Data Source

**Figure 1.**
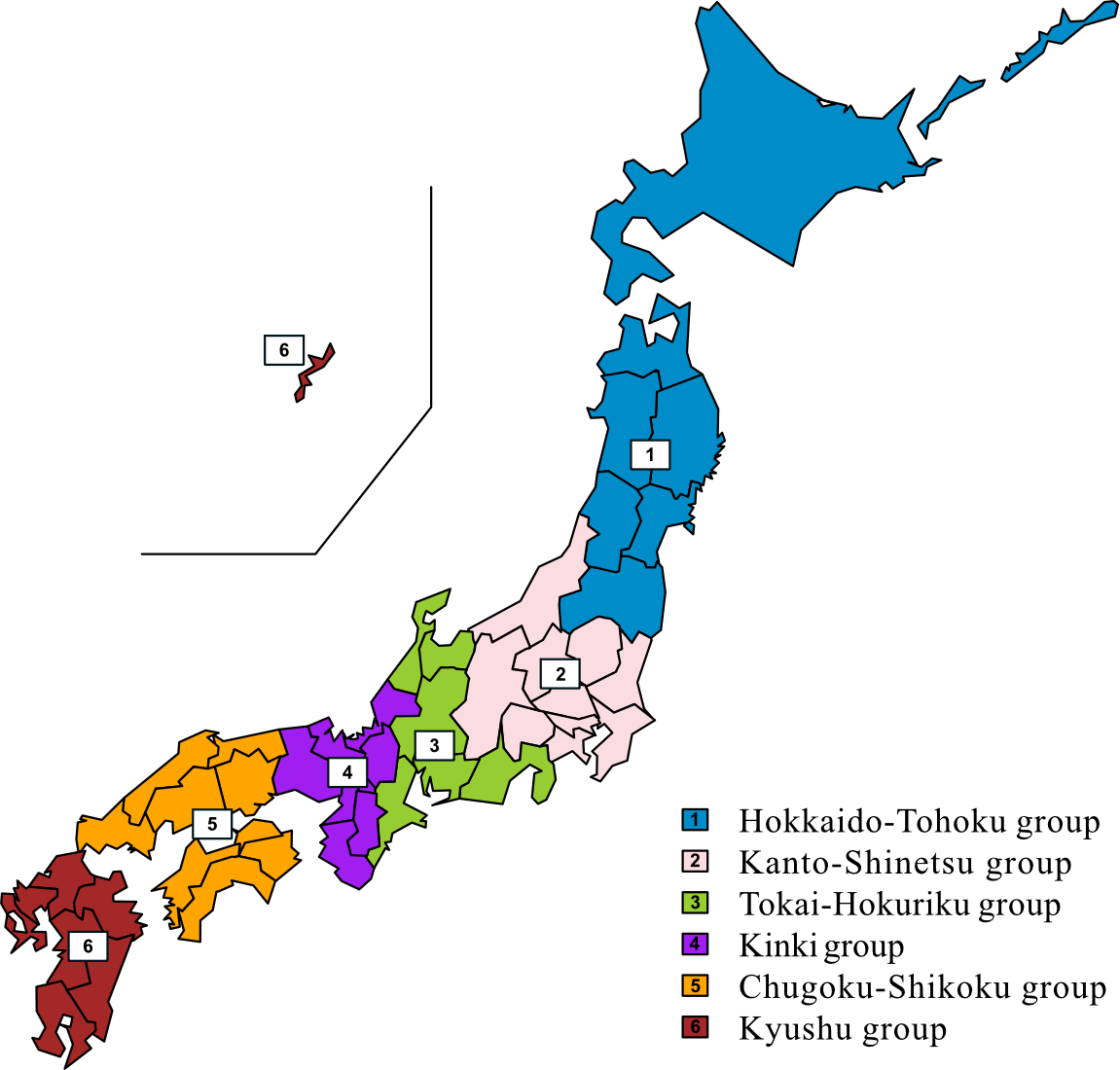
A total of 6 National Hospital Organization regional groups across Japan.

We conducted a retrospective cohort study to develop machine-learning models for predicting postoperative functional disability and in-hospital mortality in older patients with cancer. This study used data between April 2016 and March 2023 from 70 Japanese hospitals within the National Hospital Organization (NHO) database across 6 regional groups: Hokkaido-Tohoku group, Kanto-Shinetsu group, Tokai-Hokuriku group, Kinki group, Chugoku-Shikoku group, and Kyushu group (Figure 1) [18].

The NHO maintains 2 databases: (1) an administrative claims database based on the Diagnosis Procedure Combination–based Per-Diem Payment System [19] and a clinical information database based on the standardized structured medical record information exchange [20]. The administrative claims database contains patient information, such as age, sex, cost, comorbidities, complications, diagnosis, medical procedures, and medications. The clinical information database includes medical charts, laboratory data, and vital signs on a daily basis.

### Participants

The study included patients aged ≥65 years who were admitted to NHO hospitals between April 2016 and March 2023 and underwent surgery for major cancers, including lung, stomach, colorectal, liver, pancreatic, breast, and prostate cancers. These cancer sites were selected because of their high incidence [2] and mortality rates [21] in Japanese and global cancer statistics [1]. The surgical procedures included both scopic and open surgeries under general anesthesia.

We excluded patients who had missing Barthel Index data at admission or discharge, were first included in the database at admission (no medical history available), underwent surgery more than 1 week after admission, or had the Barthel Index of 0 at admission. These exclusion criteria were applied because: (1) patients missing Barthel Index data could not be evaluated for outcome, (2) most of the predictor variables were missing if patients had no medical history, (3) we eliminated the effect of hospitalization on physical function from admission to surgery, and (4) the Barthel Index change from admission to discharge was an outcome variable, but patients with a minimum score of 0 at admission cannot show further decline [22].

### Outcome Variables

The primary outcome was worse discharge, defined as either in-hospital death or postoperative functional disability. Postoperative functional disability was characterized as hospital-associated disability [16] (≥5-point decrease in the Barthel Index between admission and discharge) [17]. The Barthel Index consists of 10 items, including transfer, bathing, and stair climbing, used to evaluate activities of daily living on a scale of 0‐100, with lower scores indicating a decline in physical function.

The secondary outcomes were health care costs and postoperative length of stay (LOS) in patients predicted to be at high risk and low risk for worse discharge. The optimal cut-off point for high-risk or low-risk classification was determined using the Youden index [23] on the receiver operating characteristic (ROC) curve.

### Predictor Variables

We identified the following 37 potential predictors of worse discharge based on previous studies: age (65‐74, 75‐84, and ≥85 years) [9], sex [24], underweight (BMI <18.5 kg/m^2^) [12] and obesity (BMI ≥30 kg/m^2^) [11], route of admission (home or nonhome), emergency admission, an estimated household income in the lowest tertile based on post-code (low income) [6], smoking (Brinkman Index ≥200) [14], functional dependence (Barthel Index ≤60) [22], surgical factors (open surgery, scopic surgery, and combined general and epidural anesthesia), the presence of gastrointestinal cancer (colorectal, liver, pancreas, and stomach), the cancer staging (0–II or III–IV) [25], the presence of recurrent cancer, Charlson Comorbidity Index (CCI) ≥3 [26,27], Hospital Frailty Risk Score (HFRS) ≥5 [28], comorbidities common in older patients (cerebrovascular disease, chronic pulmonary disease, congestive heart failure, dementia [8], diabetes, liver disease, myocardial infarction, peptic ulcer disease, peripheral vascular disease, and renal disease), medical history about cancer within 8 weeks before surgery that allowed the evaluation of preoperative chemotherapy [29] and radiotherapy [30] (chemotherapy, radiation, and surgery), vital signs (body temperature ≥38°C [31], systolic blood pressure >180 mm Hg [32]), and laboratory test values (albumin <3.5 g/dL [13], total bilirubin ≥2.0 mg/dL [33], creatinine ≥ 2.0 mg/dL [33], platelet <10^5^/μL [33], and hemoglobin <11 g/dL [10]).

These predictors were measured as follows: age, sex, BMI, route of admission, emergency admission, income, cancer staging evaluated using the Tumor Nodes Metastasis (TNM) classification system [34], the presence of recurrent cancer, Brinkman Index, and Barthel Index were assessed at admission. Surgical factors were assessed during the surgery. The type of cancer was determined during the surgery using the *International Statistical Classification of Diseases and Related Health Problems, 10th Revision* (*ICD-10*) coding system [35]. Comorbidities were identified from *ICD-10* codes within 8 weeks before surgery (Table S1 in Multimedia Appendix 1). Notably, dementia was determined if it was either identified from *ICD-10* codes within 8 weeks before surgery or documented in the clinical summary at admission, as Japanese hospitals are required to include the dementia status of inpatients aged ≥65 years at admission [8]. Vital signs were measured closest to surgery after admission, and laboratory values were obtained using those measured closest to surgery within 8 weeks before surgery.

### Statistical Analysis

We randomly selected one of the 6 NHO regional groups for the external validation set [36], while hospital data from the remaining 5 groups were randomly divided into the training (70%) and the internal validation (30%) sets [37]. The missing predictor variables were imputed using the “missRanger” [38], which is a random forest–based algorithm [39], assuming that the data are missing at random.

To summarize patient characteristics, continuous variables were expressed as mean (SD) or median (IQR), depending on the distribution of variables. The Wilcoxon rank-sum test or the Welch test was used to assess between-group differences. Categorical variables were expressed as proportions and compared using the *χ*^2^ test.

In the training set, a double penalty was implemented by eliminating unnecessary variables to create a more practical model for clinical use [40]. The first penalty involved selecting predictor candidates with a crude odds ratio (OR) at *P*<.1 [41]. The second penalty to further narrow down the predictor candidates used the least absolute shrinkage and selection operator (Lasso) method, which allowed for the selection of clinically relevant variables with consistent relationships [36,42].

The selected factors were incorporated into 6 machine learning models: category boosting (CatBoost) [36,43], extreme gradient boosting (XGBoost) [36,42], logistic regression, neural networks [44], random forest [36], and support vector machine (SVM) [36]. Model performance was evaluated using the area under the ROC curve (AUC) with 95% CIs). Similarly, we calculated the accuracy, sensitivity, specificity, *F*_1_-score, and the area under the precision-recall curve (PRAUC) to assess the performance of the models [36,37]. The precision-recall curve of the models was also shown.

We used the synthetic minority oversampling and random undersampling techniques to avoid overfitting owing to the imbalance between the positive and negative events [45]. The minority class was oversampled at 50%, 100%, and 200% of its original size, followed by random undersampling of the majority class to achieve equal numbers between classes. The models were trained using 10-fold cross-validation with grid search for hyperparameter optimization. Of these sampling ratios and hyperparameter combinations, those yielding the largest AUC were selected. We ranked the predictor variable using the Shapley additive explanations (SHAP) method [46] to assess the contribution of the predictors to the models. Moreover, considering higher cancer stages are associated with poorer postoperative outcomes [25], a multiple logistic regression was conducted to evaluate the interaction between cancer staging and other predictor variables using OR (95% CI). We examined interactions between cancer stage III–IV and the top 5 features based on the mean absolute SHAP value.

We analyzed the AUC difference between the models using the DeLong method [47], which was based on the model with the largest AUC in both internal and external validation. Sensitivity analyses were used to assess the impact of missing data and assessing 2 outcomes simultaneously (death or functional disability) on the models. For missing values, a complete case analysis was performed to confirm the robustness of the results obtained from the imputed data set. We also evaluated the AUC of the models when the models predicted only death in all patients or functional disability in patients with survival discharge. We conducted subgroup analyses by LOS, cancer type (breast, colorectal, liver, lung, pancreas, prostate, or stomach), and cancer staging (stage 0–I, II, III, or IV). For the LOS analysis, we calculated the 75th percentile of LOS for each cancer type separately and divided patients into LOS for each cancer type <75th percentile (short-stay) and LOS ≥75th percentile (long-stay) groups [48].

For the analysis of secondary outcomes, we compared LOS and health care costs between patients at high and low risk based on the model with the highest AUC in both internal and external validation. Health care costs were assessed across various categories, including total, medical consultation, medication, medical procedure, surgical procedure, laboratory tests, hospital stay, and others. The currency conversion rate was 150 JPY to US $1.

All hypothesis tests had a 2-sided significance level of .05. All statistical analyses were performed using R version 4.3.1 (R Foundation for Statistical Computing) .

### Ethical Considerations

Our study was approved by the Institutional Review Board of Showa University (approval number 2023‐129-A). Individual consent was not required because this was an opt-out study. This study conforms to the principles outlined in the Transparent Reporting of a multivariable prediction model for Individual Prognosis or Diagnosis (TRIPOD) statement.

## Results

**Figure 2.**
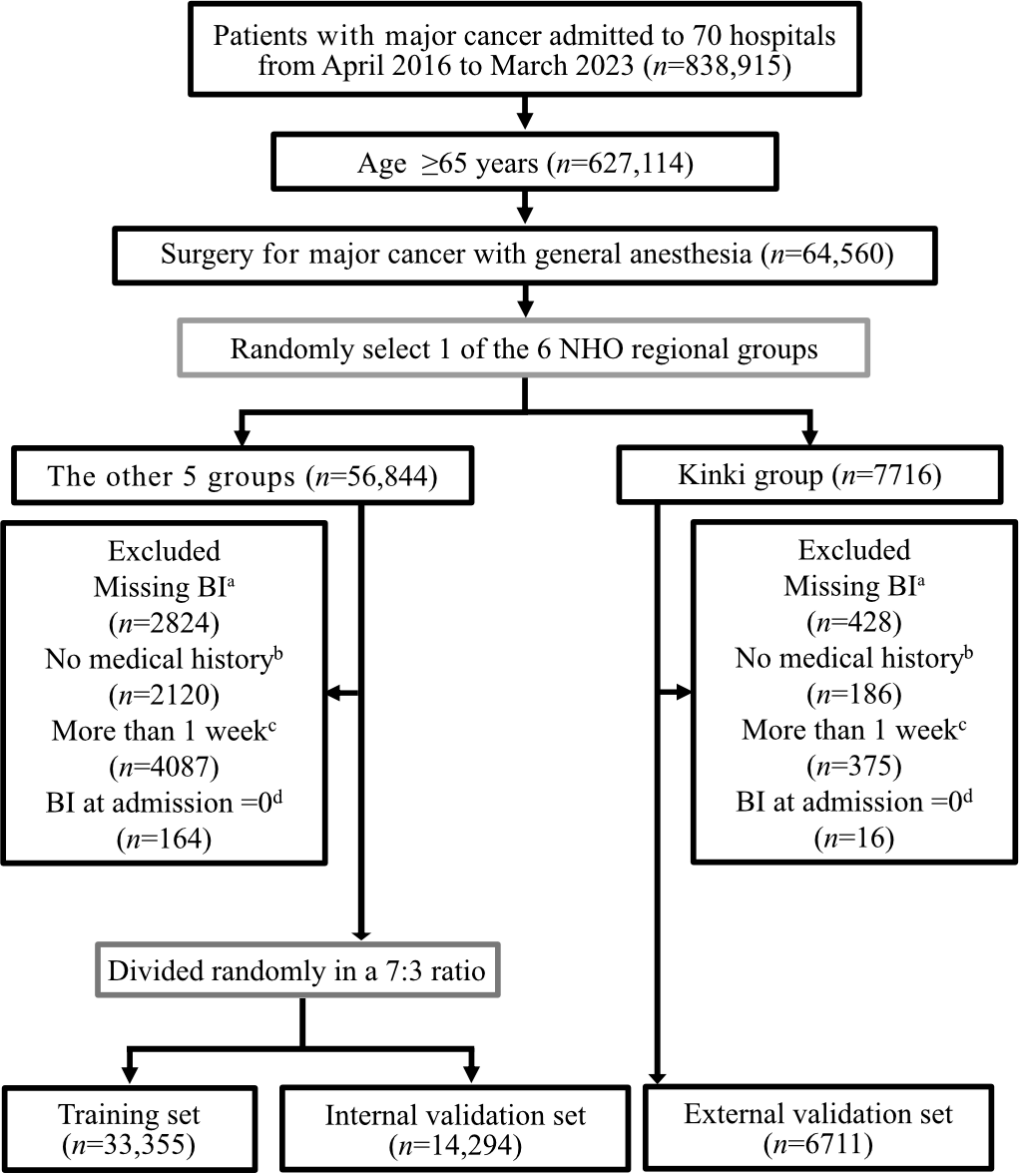
Flow diagram of enrollment of study participants.(a) “Missing BI” means patients with missing Barthel Index at admission or discharge. (b) “No medical history” means patients included in the database within 8 weeks preceding surgery. (c) “More than 1 week” means patients who underwent surgery more than one week after admission. (d) “BI at admission=0” means patients with BI of 0 at admission. BI: Barthel Index; NHO: National Hospital Organization.

In total, 54,360 patients from 70 hospitals were included in this study (Figure 2): 6711 in the external validation set from Kinki group, 33,355 in the training set, and 14,294 in the internal validation set from the remaining 5 regional groups. In the training set, 1406/33,355 (4.2%) patients experienced worse discharge (Table 1). These patients were older (24.5% [344/1406] vs 5.8% [1864/31,949] for age ≥85 years), more likely to be admitted from nonhome (8.1% [114/1406] vs 0.9% [281/31,949]), and had higher prevalence of dementia (28.7% [403/1406] vs 5.6% [1783/31,949]), gastrointestinal cancer (64.3% [904/1406] vs 41.7% [13,326/31,949]), and advanced cancer stage (32.6% [458/1406] vs 24.2% [7716/31,9] for stage III–IV).

In the training set, we selected 31 predictor variables for worse discharge using crude OR (Table S2 in Multimedia Appendix 1). Further selection using the Lasso method resulted in 24 factors: age (75‐84 y and ≥85 y), male sex, BMI <18.5 kg/m^2^, nonhome admission, emergency admission, low income, Barthel Index at admission ≤60, open surgery, gastrointestinal cancer, cancer stage III–IV, HFRS ≥5, cerebrovascular disease, congestive heart failure, dementia, diabetes, liver disease, myocardial infarction, medical history of chemotherapy, systolic blood pressure ≥180 mm Hg, albumin<3.5 g/dL, creatinine ≥ 2.0 mg/dL, platelet <10^5^/μL, and hemoglobin <11 g/dL. All 6 models were developed using these 24 variables.

**Figure 3.**
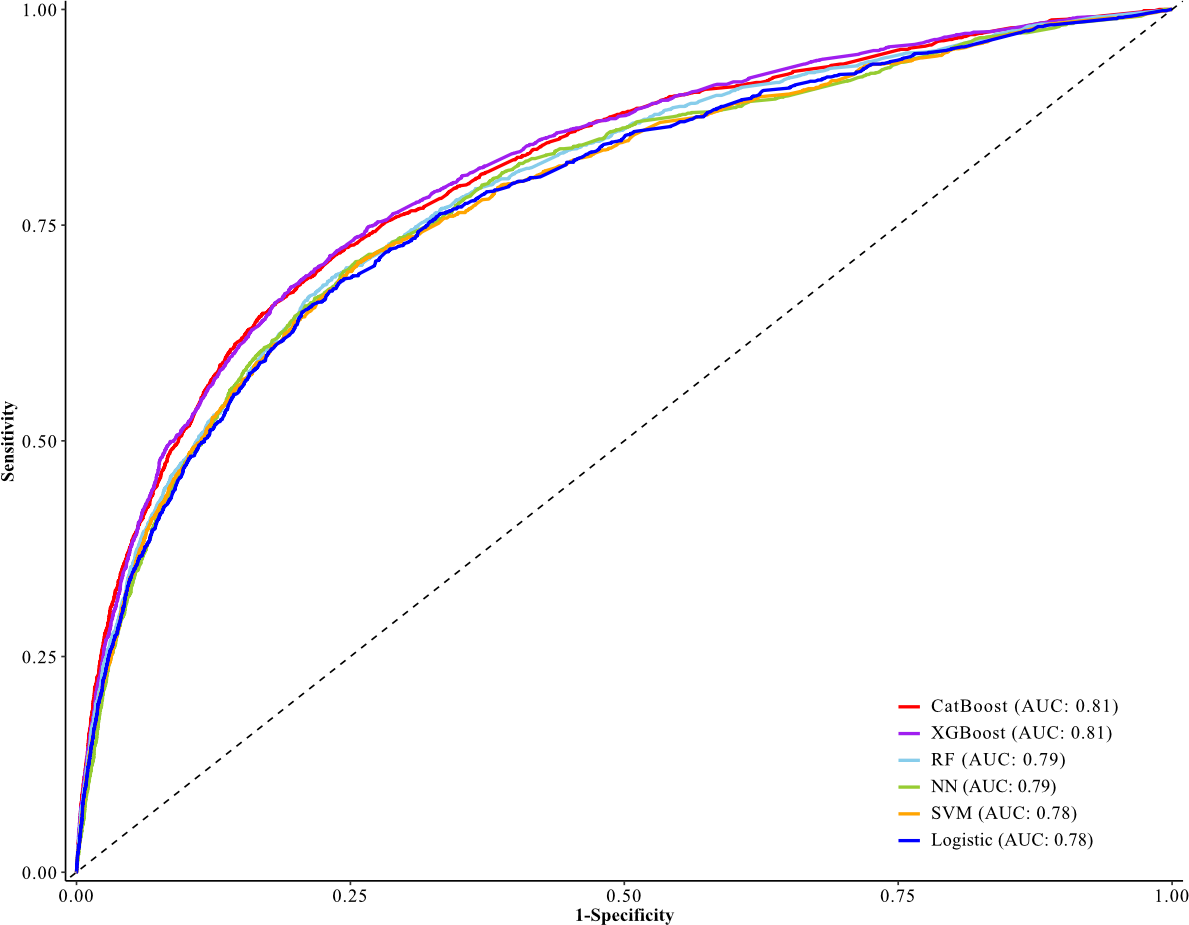
Receiver operating characteristic curves of 6 machine-learning models in the training set. AUC: area under the receiver operating characteristic curve; CatBoost: category boosting; Logistic: logistic regression; NN: neural networks; RF: random forest; SVM: support vector machine; XGBoost: extreme gradient boosting.

**Figure 4.**
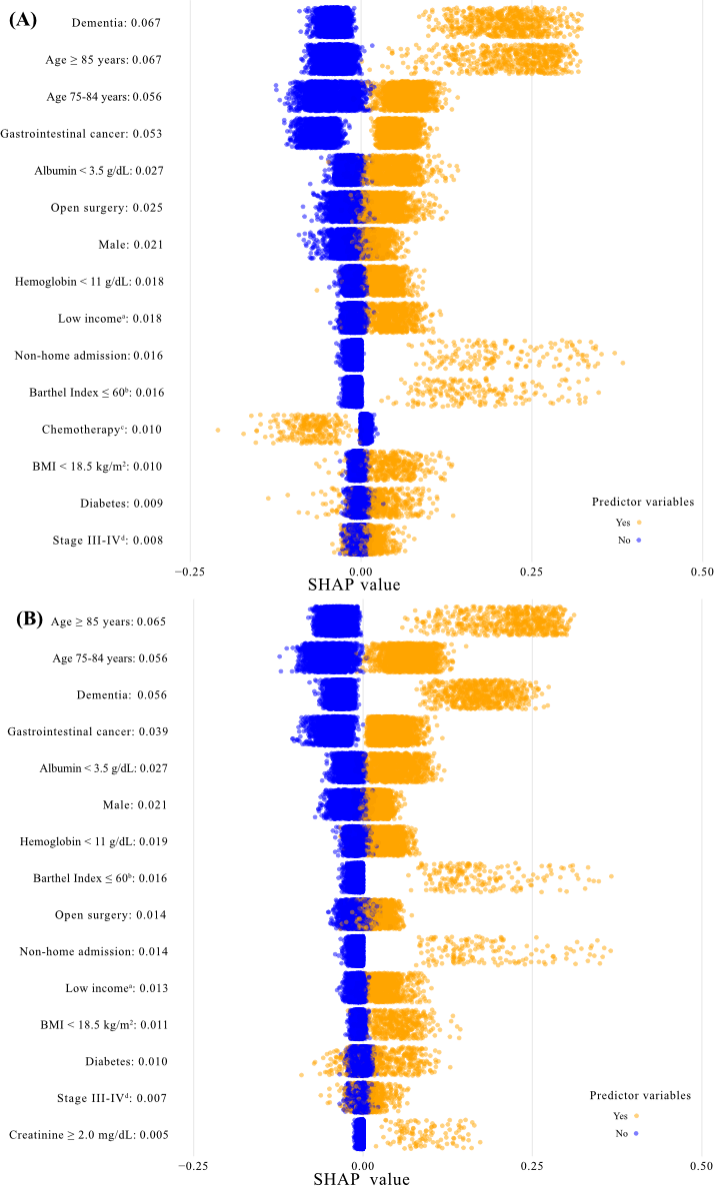
The top 15 features of predictor variables based on mean absolute Shapley additive explanations value. (**A**) The top 15 features selected by the category boosting model. (**B**) The top 15 features selected by the XGBoost: extreme gradient boosting model. (a) “Low income” means an estimated household income in the lowest tertile based on ZIP code. (b) “Barthel Index ≤60” means the Barthel Index ≤60 at admission. (c) “Chemotherapy” means that patients underwent chemotherapy within 8 weeks before surgery. (d) “Stage III-IV” means that patients had cancer staging III or IV using the TNM: Tumor Nodes Metastasis classification system.CatBoost: category boosting; SHAP: Shapley additive explanations; XGBoost: extreme gradient boosting.

Furthermore, the AUCs were 0.81 (95% CI 0.80‐0.82) for CatBoost, 0.81 (95% CI 0.80‐0.82) for XGBoost, 0.79 (95% CI 0.78‐0.80) for random forest, 0.79 (95% CI 0.78‐0.80) for neural networks, 0.78 (95% CI 0.77‐0.80) for SVM, and 0.78 (95% CI 0.77‐0.80) for logistic regression (Figure 3). CatBoost and XGBoost were the 2 models with AUC ≥0.80 and showed similar values with relatively high accuracy (0.76), sensitivity (0.72), specificity (0.76), *F*_1_-score (0.20), and area under the precision-recall curve (PRAUC) (0.22). The performance metrics and precision-recall curves for all models are shown in Table S3 and Figure S1 in Multimedia Appendix 1, respectively. In the top 15 influential factors based on the mean absolute SHAP value, the CatBoost and XGBoost models had the same combination for the 14 features: dementia, age ≥85 years, age 74‐85 years, gastrointestinal cancer, albumin <3.5 g/dL, open surgery, male sex, hemoglobin <11 g/dL, low income, nonhome admission, Barthel Index at admission ≤60, BMI <18.5 kg/m^2^, diabetes, and stage III–IV (Figure 4). There were no significant interactions between stage III–IV and the top 5 influential features that both models shared (Table S4) in Multimedia Appendix 1.

**Figure 5.**
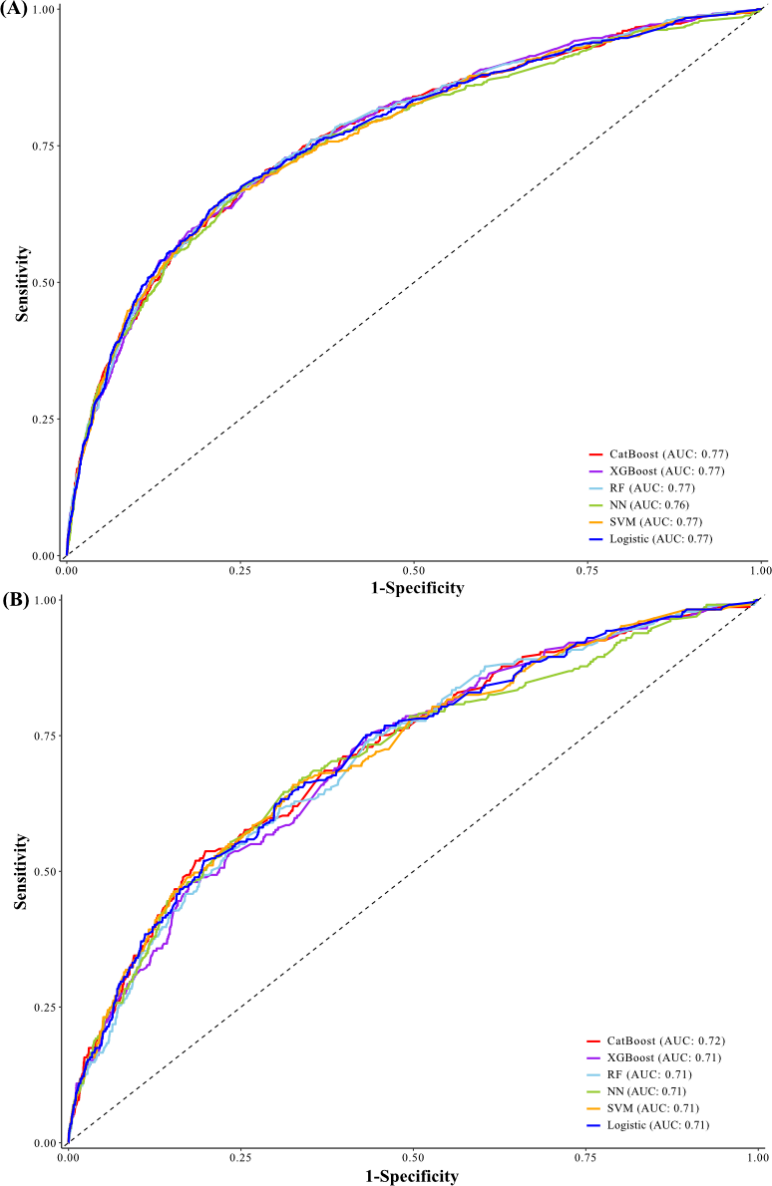
Receiver operating characteristic curves of 6 machine learning models. (**A**) Receiver operating characteristic curves in the internal validation set. (**B**) Receiver operating characteristic curves in the external validation set. AUC: area under the receiver operating characteristic curve; CatBoost: category boosting; Logistic: logistic regression; NN: neural networks; RF: random forest; ROC: receiver operating characteristic; SVM: support vector machine; XGBoost: extreme gradient boosting.

For both the internal and external validation set, the CatBoost model had the largest AUCs among the 6 machine- learning models: 0.77 (95% CI 0.75‐0.79) and 0.72 (95% CI 0.68‐0.75), respectively (Figure 5). In sensitivity analysis, all 6 models maintained comparable performance to the main analysis (Table S5 in Multimedia Appendix 1). The CatBoost model achieved relatively high AUCs and showed consistent performance in complete cases (0.78, 95% CI 0.76‐0.80; 0.72, 0.68‐0.76), death only (0.77, 0.71‐0.82; 0.73, 0.65‐0.81), and functional disability only (0.77, 0.75‐0.79; 0.71, 0.68‐0.75) for internal and external validation, respectively.

In subgroup analyses, the models maintained consistent performance for LOS and cancer staging (Table S5 in Multimedia Appendix 1). However, the performance varied based on cancer types: the CatBoost model achieved a larger AUC in patients with stomach cancer (internal: 0.80, 95% CI 0.76‐0.84; external: 0.81, 95% CI 0.69‐0.92) but a smaller AUC in patients with prostate cancer (0.53, 95% CI 0.40‐0.66; 0.46, 95% CI 0.28‐0.63) than the main analysis.

Based on the CatBoost model, patients at high risk had significantly longer LOS (internal: median 13, IQR 9‐19 d vs median 9, IQR 7‐13 d; external: median 13, IQR 10‐19 d vs median 10.0, IQR 7.0‐14.0 d) and higher total health care costs (internal: median US $11,048, IQR US $9191‐13,106 d vs median US $10,092, IQR US $7894‐11,893; external: median 11,069, IQR US $9401‐13,499 vs median US $10,371, IQR US $8820‐11,936) than patients at low risk (all *P*<.01). However, the high-risk group had slightly lower surgical procedure costs than the low-risk group in internal validation and was comparable to the low-risk group in external validation (Table 2).

**Table 1.** Patient background with or without worse discharge in the training set.

Variable	No worse discharge (n=31,949)	Worse discharge (n=1406)	*P* value
Age (years), n (%)			<.01
65‐74	18,010 (56.4)	405 (28.8)	
75‐84	12,075 (37.8)	657 (46.7)	
≥85	1864 (5.8)	344 (24.5)	
Sex, n (%)			.05
Male	16,570 (51.9)	768 (54.6)	
Female	15,379 (48.1)	638 (45.4)	
BMI (kg/m^2^), n (%)			
<18.5	2535 (7.9)	212 (15.1)	<.01
≥30	1170 (3.7)	57 (4.1)	.49
Route of admission, n (%)			
Home	31,668 (99.1)	1292 (91.9)	<.01
Nonhome	281 (0.9)	114 (8.1)	<.01
Nursing home	134 (0.4)	60 (4.3)	<.01
Other hospital	136 (0.4)	54 (3.8)	<.01
Others	11 (0)	0 (0)	1.00
Emergency admission, n (%)	252 (0.8)	42 (3)	<.01
Low income^a^, n (%)	6020 (18.8)	352 (25)	<.01
Brinkman Index ≥200, n (%)	13,307 (41.7)	551 (39.2)	.07
Barthel Index^b^≤60, n (%)	422 (1.3)	139 (9.9)	<.01
Open surgery, n (%)	12,307 (38.5)	652 (46.4)	<.01
Scopic surgery, n (%)	19,642 (61.5)	754 (53.6)	.01
With epidural anesthesia^c^, n (%)	15,742 (49.3)	769 (54.7)	<.01
Type of cancer, n (%)			<.01
Breast	6298 (19.7)	150 (10.7)	
Colorectal	7387 (23.1)	493 (35.1)	
Liver	1331 (4.2)	91 (6.5)	
Lung	9386 (29.4)	293 (20.8)	
Pancreas	786 (2.5)	62 (4.4)	
Prostate	2939 (9.2)	59 (4.2)	
Stomach	3822 (12)	258 (18.3)	
Gastrointestinal cancer	13,326 (41.7)	904 (64.3)	<.01
Cancer staging, n (%)			<.01
0-I	14,398 (45.1)	492 (35)	
II	9835 (30.8)	456 (32.4)	
III	4845 (15.2)	316 (22.5)	
IV	2871 (9)	142 (10.1)	
Stage III-IV	7716 (24.2)	458 (32.6)	<.01
Recurrent cancer, n (%)	2161 (6.8)	82 (5.8)	.19
Comorbidities, n (%)			
CCI^d^ ≥3	4508 (14.1)	227 (16.1)	.04
HFRS^e^ ≥5	270 (0.8)	55 (3.9)	<.01
Cerebrovascular disease	853 (2.7)	87 (6.2)	<.01
Chronic pulmonary disease	1832 (5.7)	95 (6.8)	.12
Congestive heart failure	823 (2.6)	72 (5.1)	<.01
Dementia	1783 (5.6)	403 (28.7)	<.01
Diabetes	4122 (12.9)	232 (16.5)	<.01
Liver disease	987 (3.1)	66 (4.7)	<.01
Myocardial infarction	223 (0.7)	17 (1.2)	.04
Peptic ulcer disease	1597 (5)	85 (6)	.09
Peripheral vascular disease	195 (0.6)	16 (1.1)	.02
Renal disease	341 (1.1)	27 (1.9)	<.01
Medical history within 8 weeks, n (%)			
Chemotherapy	2573 (8.1)	88 (6.3)	.02
Radiation	75 (0.2)	6 (0.4)	.25
Surgery	4220 (13.2)	215 (15.3)	.03
BT^f^ ≥38˚C, n (%)	4212 (13.2)	190 (13.5)	.75
sBP^g^ ≥180 mm Hg, n (%)	1898 (5.9)	126 (9)	<.01
Albumin <3.5 g/dL, n (%)	6669 (20.9)	543 (38.6)	<.01
T-Bil^h^ ≥2.0 mg/dL, n (%)	252 (0.8)	14 (1)	.48
Creatinine ≥2.0 mg/dL, n (%)	639 (2)	65 (4.6)	<.01
Platelet <10^5^/μL, n (%)	641 (2)	59 (4.2)	<.01
Hemoglobin <11 g/dL, n (%)	5896 (18.5)	523 (37.2)	<.01
Number of beds, n (%)			<.01
<300	1298 (4.1)	70 (5)	
300‐499	17,922 (56.1)	707 (50.3)	
≥500	12,729 (39.8)	629 (44.7)	

a Low income” means an estimated household income in the lowest tertile based on ZIP code.

b Barthel Index ≤ 60” means the Barthel Index ≤ 60 at admission.

c With epidural anesthesia” means the combination of general and epidural anesthesia.

d CCI: Charlson Comorbidity Index.

e HFRS: Hospital Frailty Risk Score.

f BT: body temperature.

g sBP: systolic blood pressure.

h T-Bil: total bilirubin.

**Table 2.** Adverse outcome between low-risk and high-risk group by category boosting model using the Youden index as cut-off value.

Variable	Internal validation set			External validation set		
	Low risk (n=10,788)	High risk (n=3506)	*P* value	Low risk (n=5459)	High risk (n*=*1252)	*P* value
Worse discharge, n (%)	221 (2)	386 (11)	<.01	116 (2.1)	113 (9)	<.01
Death, n (%)	25 (0.2)	33 (0.9)	<.01	16 (0.3)	18 (1.4)	<.01
Functional disability, n (%)^a^	196 (1.8)	353 (10.1)	<.01	100 (1.8)	95 (7.6)	<.01
LOS^b^, median (IQR), days	9 (7-13)	13.0 (9-19)	<.01	10 (7-14)	13 (10-19)	<.01
Cost, median (IQR), US $						
Total	10,092 (7894-11,893)	11,048 (9191-13,106)	<.01	10,371 (8820-11,936)	11,069 (9401-13,499)	<.01
Medical consultation	77 (49-114)	97 (65-141)	<.01	75 (48-110)	97 (70-137)	<.01
Medication	68 (33, 139)	176 (87-362)	<.01	67 (34-139)	146 (76-333)	<.01
Medical procedure	26 (15-42)	40 (24-67)	<.01	27 (14-44)	28 (13-57)	<.01
Surgical procedure	6645 (5028-8253)	6620 (5513-8116)	.02	7203 (5825-8176)	6924 (5900-8212)	.46
Laboratory tests	449 (328-597)	516 (370-742)	<.01	443 (328-610)	526 (371-775)	<.01
Hospital stays	2404 (1873-3021)	3085 (2362-4073)	<.01	2484 (1940-3078)	3039 (2419-4178)	<.01
Others	0 (0-137)	100 (0-212)	<.01	0 (0-120)	0 (0-177)	<.01

a Functional disability” means a decrease in the Barthel Index by ≥5 points at discharge compared with admission.

b LOS: length of stay.

## Discussion

### Principal Findings

In this study, we developed and validated machine-learning models to predict postoperative functional disability and mortality in older patients with cancer. Our CatBoost model achieved good performance using routinely available preoperative factors from electronic health records, indicating the potential for clinical implementation. Although ethical training for hospital staff is essential to prevent unauthorized disclosure of prediction results, implementing this model within closed electronic health record systems could provide protection for patient privacy.

The previous model for lower-extremity surgery had an AUC of 0.72 in external validation [6], similar to our model; however, our model directly predicted functional disability using the Barthel Index rather than using nursing home discharge as a surrogate. The model performance remained consistent across sensitivity analyses for death and functional disability separately, and complete cases, indicating the robustness of our findings. Notably, the model demonstrated higher predictive performance in patients with stomach or colorectal cancer than the other cancers, making it especially valuable for surgical decision-making in these patients.

The CatBoost model identified patients at high risk who had longer LOS and higher health care costs; however, surgical procedure costs were comparable between patients at high and low risk. These findings suggested that the increased cost was based on the varying postoperative course. Our model can support decision-making for older patients with cancer and their families regarding cancer surgery by providing insights into potential postoperative QOL and care burden. Moreover, if patients at high risk choose to undergo cancer surgery, our model may enable health care providers to implement targeted interventions such as intensive postoperative rehabilitation. Early identification of patients at high risk, such as those aged ≥85 years with dementia, can help health care providers prepare support systems, including caregiver education, social work consultation for home health support, and coordination with multidisciplinary teams [15]. This proactive approach may help reduce caregiver burden and improve outcomes for both patients and their families.

Of the 6 machine-learning models, the CatBoost and XGBoost models, with AUC ≥0.80, had the same combination of 14 features in the top 15 influential factors. These factors include established risk factors for poor postoperative outcomes in older patients as identified in previous studies: dementia [7], older age [9] (≥85 y and 75‐84 y), male sex [24], anemia [10] (hemoglobin <11 g/dL), low income [6], underweight [12] (BMI <18.5 kg/m²), diabetes [49], and cancer staging [25]. In addition, several factors serve as proxies for known risk factors. For frailty [5], the factors include (1) open surgery, which generally results in a more pronounced postoperative functional disability compared with scopic surgeries; (2) nonhome admission, likely indicating that patients are too frail to live independently; and (3) Barthel Index ≤60 at admission, indicating severe dependence [22]. For malnutrition [13], the proxies are (1) albumin <3.5 g/dL, a marker of malnutrition, and (2) gastrointestinal cancer, which often involves a long time to restart food intake after surgery, increasing the risk of malnutrition compared with other cancer types. The consistency between the 2 models in identifying these factors further validates their importance in predicting postoperative outcomes. Although chemotherapy and creatinine ≥2.0 mg/dL were not common in both models, these factors, identified as influential factors in previous studies [33,50], might also be included as predictor variables in future models. Moreover, our models included several factors associated with social vulnerability, such as age ≥85 years, dementia, and low income. Without ensuring model transparency to health care providers, our model could unconsciously contribute to reduced surgical care access for vulnerable populations. Therefore, when implementing our model in clinical practice, health care providers should consider these model characteristics to ensure fair allocation of health care resources [51].

### Limitations

Our study has some limitations. First, we only validated all models using Japanese data. While our CatBoost model showed moderate accuracy (AUC: 0.7‐0.9) [52] in both internal and external validation, the AUC in the external validation was lower than that in the internal validation as observed in a previous study [6]. Further studies using data from other countries and ethnic groups are necessary to evaluate model robustness, including potential bias and overfitting [51], and confirm their applicability to different health care systems. However, our study used data from 70 hospitals across Japan, which may enhance generalizability within the country. In addition, considering the global trend of population aging, our models may prove valuable for other countries in the future, particularly when these countries reach levels of demographic aging similar to Japan’s current situation.

Second, we did not have information on predictor variables such as marital status [6] because of the retrospective nature of the study. Despite this limitation, our models had good predictive performance in the validation sets. While our analysis showed no significant interactions between stage III–IV cancer and the top features, future studies incorporating additional variables may evaluate such interactions to enhance the predictive performance of models.

Finally, the long-term prognosis of patients classified as high-risk by our models remains unclear. Further research is required to determine the extent of functional recovery and mortality in these patients. At a minimum, postoperative functional disability in patients at high risk indicates an increased immediate post-discharge burden on family caregivers and health care resources.

### Conclusions

Our CatBoost model achieved good performance for predicting postoperative functional disability and mortality in older patients with cancer. This model could support surgical decision-making for patients and families while guiding targeted interventions by health care providers. This model, which is based on routinely available preoperative factors, has the potential for implementation in clinical settings through electronic health records.

## Supplementary material

10.2196/65898 Multimedia Appendix 1 Table S1. ICD-10 codes selected as predictor variables. Table S2. Crude odds ratios of predictor variables for worse discharge. Table S3. Performance metrics of six machine learning models in training set. Table S4. Interaction between stage III–IV and top 5 features of predictor variables based on mean absolute SHAP value in training set. Table S5. AUCs of six machine learning models for internal and external validation set in sensitivity and subgroup analyses. Figure S1. Precision-recall curve of six machine learning models in training set.

## Abbreviations

AUC: area under the receiver operating characteristic curve
CatBoost: category boosting
CCI: Charlson Comorbidity Index
HFRS: Hospital Frailty Risk Score
*ICD-10*: *International Statistical Classification of Diseases and Related Health Problems, 10th Revision*
Lasso: least absolute shrinkage and selection operator
LOS: length of stay
NHO: National Hospital Organization
OR: odds ratio
PRAUC: area under the precision-recall curve
QOL: quality of life
ROC: receiver operating characteristic
SHAP: Shapley additive explanations
TNM: Tumor Node Metastasis
TRIPOD: Transparent Reporting of a multivariable prediction model for Individual Prognosis or Diagnosis
XGBoost: extreme gradient boosting
