# Supplementary material for: Machine Learning for Predicting Postoperative Functional Disability and Mortality Among Older Patients With Cancer: Retrospective Cohort Study
Source: JMIR Aging. 2025 May 14;8:e65898. doi: 10.2196/65898 (PMC12094529; doi:10.2196/65898)
Supplement: Multimedia Appendix 1 [file aging-v8-e65898-s001.docx]

**Multimedia Appendix**

**Table S1.** ICD-10 codes selected as predictor variables.

**Table S2.** Crude odds ratios of predictor variables for worse discharge.

**Table S3.** Performance metrics of six machine learning models in training set.

**Table S4.** Interaction between stage III–IV and top 5 features of predictor variables based on mean absolute SHAP value in training set.

**Table S5.** AUCs of six machine learning models for internal and external validation set in sensitivity and subgroup analyses.

**Figure S1.** Precision-recall curve of six machine learning models in training set.

Table S1. ICD-10 codes selected as predictor variables.

| Disease | ICD-10 codes |
| --- | --- |
|  |  |
| Type of cancer |  |
| Breast | C500, C501, C502, C503, C504, C505, C506, C508, C509 |
| Lung | C340, C341, C342, C343, C348, C349, C780 |
| Prostate | C61 |
| Colorectal | C180, C181, C182, C183, C184, C185, C186, C187, C189, C19, C20, C785 |
| Liver | C220, C221, C222, C223, C224, C227, C229, C787 |
| Pancreas | C250, C251, C252, C253, C254, C257, C258, C259 |
| Stomach | C160, C161, C162, C163, C164, C165, C166, C169 |
| Metastatic cancer | C77, C78, C79, C80 |
| Comorbidities |  |
| Cerebrovascular disease | G45, G46, H340, I60, I61, I62, I63, I64, I65, I66, I67, I68, I69 |
| Chronic pulmonary disease | I278, I279, J40, J41, J42, J43, J44, J45, J46, J47, J60, J61, J62, J63, J64, J65, J66, J67, J684, J701, J703 |
| Congestive heart failure | I099, I110, I130, I132, I255, I420, I425, I426, I427, I428, I429, I43, I50, P290 |
| Dementia | F00, F01, F02, F03, F051, G30, G311 |
| Diabetes | E100, E101, E102, E103, E104, E105, E106, E107, E108, E109, E110, E111, E112, E113, E114, E115, E116, E117, E118, E119, E120, E121, E122, E123, E124, E125, E126, E127, E128, E129, E130, E131, E132, E133, E134, E135, E136, E137, E138, E139, E140, E141, E142, E143, E144, E145, E146, E147, E148, E149 |
| Liver disease | B18, I850, I859, I864, I982, K700, K701, K702, K703, K704, K709, K711, K713, K714, K715, K717, K721, K729, K73, K74, K760, K762, K763, K764, K765, K766, K767, K768, K769, Z944 |
| Myocardial infarction | I21, I22, I252 |
| Peptic ulcer disease | K25, K26, K27, K28 |
| Peripheral vascular disease | I70, I71, I731, I738, I739, I771, I790, I792, K551, K558, K559, Z958, Z959 |
| Renal disease | I120, I131, N032, N033, N034, N035, N036, N037, N052, N053, N054, N055, N056, N057, N18, N19, N250, Z490, Z491, Z492, Z940, Z992 |

ICD-10 = International Classification of Disease 10th Revision.

Table S2. Crude odds ratios of predictor variables for worse discharge.

| Variable | *P* | OR (95%CI) |
| --- | --- | --- |
|  |  |  |
| Dementia | < 0.001 | 6.80 (5.99, 7.70) |
| Age ≥ 85 years | < 0.001 | 5.23 (4.58, 5.95) |
| Barthel Index ≤ 60^a^ | < 0.001 | 8.20 (6.69, 9.98) |
| Non-home | < 0.001 | 9.94 (7.92, 12.41) |
| Hemoglobin < 11 g/dL | < 0.001 | 2.62 (2.34, 2.93) |
| Gastrointestinal cancer | < 0.001 | 2.52 (2.25, 2.81) |
| Albumin < 3.5 g/dL | < 0.001 | 2.39 (2.13, 2.66) |
| HFRS ≥ 5 | < 0.001 | 4.78 (3.52, 6.36) |
| BMI, mean (SD), kg/m^2^ | < 0.001 | 2.06 (1.77, 2.39) |
| Emergency admission | < 0.001 | 3.87 (2.74, 5.33) |
| Cerebrovascular disease | < 0.001 | 2.40 (1.90, 3.00) |
| Stage III-IV | < 0.001 | 1.52 (1.35, 1.70) |
| 75-84 years old | < 0.001 | 1.44 (1.30, 1.61) |
| Creatinine ≥ 2.0 mg/dL | < 0.001 | 2.38 (1.81, 3.06) |
| Open surgery | < 0.001 | 1.38 (1.24, 1.54) |
| Low income^b^ | < 0.001 | 1.44 (1.27, 1.63) |
| Congestive heart failure | < 0.001 | 2.04 (1.58, 2.59) |
| Platelet < 10^5^/μL | < 0.001 | 2.14 (1.61, 2.78) |
| sBP ≥ 180 mmHg | < 0.001 | 1.56 (1.28, 1.87) |
| With epidural anesthesia^c^ | < 0.001 | 1.24 (1.12, 1.38) |
| Diabetes | < 0.001 | 1.33 (1.15, 1.54) |
| Liver disease | < 0.001 | 1.55 (1.19, 1.98) |
| Renal disease | < 0.01 | 1.81 (1.19, 2.64) |
| Medical history of chemotherapy | 0.02 | 0.76 (0.61, 0.94) |
| Peripheral vascular disease | 0.02 | 1.87 (1.08, 3.03) |
| Medical history of surgery | 0.02 | 1.19 (1.02, 1.37) |
| Myocardial infarction | 0.03 | 1.74 (1.02, 2.77) |
| CCI ≥ 3 | 0.03 | 1.17 (1.01, 1.35) |
| Male | 0.04 | 1.12 (1.00, 1.24) |
| Brinkman Index ≥ 200 | 0.07 | 0.90 (0.81, 1.01) |
| Peptic ulcer disease | 0.08 | 1.22 (0.97, 1.52) |
| Chronic pulmonary disease | 0.11 | 1.19 (0.96, 1.47) |
| Medical history of radiation | 0.16 | 1.82 (0.71, 3.85) |
| Recurrent cancer | 0.17 | 0.85 (0.68, 1.06) |
| T-Bil ≥ 2.0 mg/dL | 0.39 | 1.27 (0.70, 2.09) |
| BMI < 18.5 kg/m2 | 0.44 | 1.11 (0.84, 1.44) |
| BT ≥ 38˚C | 0.72 | 1.03 (0.88, 1.20) |

a: “Barthel Index ≤ 60” means the Barthel Index ≤ 60 at admission.

b: “Low income” means an estimated household income in the lowest tertile based on ZIP code.

c: “With epidural anesthesia” means the combination of general and epidural anesthesia.

95%CI = 95% confidence interval; BT = body temperature; CCI = Charlson Comorbidity Index; HFRS = Hospital Frailty Risk Score; OR = odds ratio; sBP = systolic blood pressure; T-Bil = total bilirubin.

Table S3. Performance metrics of six machine learning models in training set.

| Model | AUC | Accuracy | Sensitivity | Specificity | F1 score | PRAUC |
| --- | --- | --- | --- | --- | --- | --- |
|  |  |  |  |  |  |  |
| CatBoost | **0.81** | **0.76** | **0.72** | 0.76 | **0.20** | **0.22** |
| XGBoost | **0.81** | **0.76** | **0.72** | 0.76 | **0.20** | **0.22** |
| RF | 0.79 | **0.76** | 0.69 | **0.77** | **0.20** | 0.20 |
| NN | 0.79 | 0.74 | 0.71 | 0.74 | 0.19 | 0.18 |
| SVM | 0.78 | 0.75 | 0.69 | 0.76 | 0.19 | 0.19 |
| Logistic | 0.78 | **0.76** | 0.68 | 0.76 | 0.19 | 0.19 |

AUC = the area under the receiver operating characteristic curve; CatBoost = category boosting; Logistic = logistic regression; NN = neural networks; PRAUC = area under the precision-recall curve; RF = random forest; SVM = support vector machine; XGBoost = extreme gradient boosting.

Table S4. Interaction between stage III–IV and top 5 features of predictor variables based on mean absolute SHAP value in training set.

| Predictor variables | OR (95%CI) | *P* |
| --- | --- | --- |
|  |  |  |
| Age 75–84 years | 1.01 (0.80, 1.27) | 0.43 |
| Age ≥ 85 years | 0.92 (0.69, 1.21) | 0.54 |
| Alb < 3.5 g/dL | 1.01 (0.80, 1.28) | 0.93 |
| Dementia | 0.90 (0.69, 1.17) | 0.43 |
| Gastrointestinal cancer | 0.84 (0.65, 1.08) | 0.18 |

95%CI = 95% confidence interval; OR = odds ratio; SHAP = Shapley additive explanations.

Table S5. AUCs of six machine learning models for internal and external validation set in sensitivity and subgroup analyses.

| Model | Internal validation set | | External validation set | |
| --- | --- | --- | --- | --- |
|  | AUC (95% CI) | *P* | AUC (95% CI) | *P* |
|  |  |  |  |  |
| **All cases** |  |  |  |  |
| CatBoost | 0.77 (0.75, 0.79) | - | 0.72 (0.68, 0.75) | - |
| XGBoost | 0.77 (0.75, 0.79) | 0.70 | 0.71 (0.67, 0.74) | 0.19 |
| RF | 0.77 (0.75, 0.79) | 0.60 | 0.71 (0.68, 0.74) | 0.29 |
| NN | 0.76 (0.74, 0.78) | 0.06 | 0.71 (0.67, 0.74) | 0.29 |
| SVM | 0.77 (0.75, 0.79) | 0.42 | 0.71 (0.68, 0.75) | 0.75 |
| Logistic | 0.77 (0.75, 0.79) | 0.90 | 0.71 (0.68, 0.75) | 0.81 |
| **Sensitivity analyses** |  |  |  |  |
| Complete cases |  |  |  |  |
| CatBoost | 0.78 (0.76, 0.80) | - | 0.72 (0.68, 0.76) | - |
| XGBoost | 0.78 (0.76, 0.80) | 0.71 | 0.71 (0.67, 0.75) | 0.25 |
| RF | 0.78 (0.76, 0.81) | 0.23 | 0.71 (0.67, 0.75) | 0.57 |
| NN | 0.77 (0.75, 0.80) | 0.16 | 0.72 (0.68, 0.76) | 0.80 |
| SVM | 0.78 (0.76, 0.80) | 0.90 | 0.72 (0.68, 0.76) | 0.80 |
| Logistic | 0.78 (0.76, 0.80) | 0.57 | 0.72 (0.68, 0.76) | 0.57 |
| Death |  |  |  |  |
| CatBoost | 0.77 (0.71, 0.82) | - | 0.73 (0.65, 0.81) | - |
| XGBoost | 0.78 (0.72, 0.83) | 0.33 | 0.74 (0.66, 0.82) | 0.54 |
| RF | 0.76 (0.71, 0.82) | 0.65 | 0.72 (0.65, 0.80) | 0.94 |
| NN | 0.75 (0.68, 0.81) | 0.20 | 0.72 (0.64, 0.80) | 0.88 |
| SVM | 0.77 (0.71, 0.83) | 1.00 | 0.75 (0.67, 0.83) | 0.38 |
| Logistic | 0.77 (0.71, 0.83) | 0.82 | 0.75 (0.68, 0.83) | 0.29 |
| Functional disability |  |  |  |  |
| CatBoost | 0.77 (0.75, 0.79) | - | 0.71 (0.68, 0.75) | - |
| XGBoost | 0.77 (0.75, 0.79) | 0.91 | 0.70 (0.66, 0.74) | 0.10 |
| RF | 0.77 (0.75, 0.79) | 0.46 | 0.71 (0.67, 0.74) | 0.26 |
| NN | 0.76 (0.74, 0.78) | 0.13 | 0.70 (0.66, 0.74) | 0.27 |
| SVM | 0.77 (0.74, 0.79) | 0.40 | 0.71 (0.67, 0.75) | 0.37 |
| Logistic | 0.77 (0.75, 0.79) | 0.98 | 0.71 (0.67, 0.75) | 0.38 |
| **Subgroup analyses** |  |  |  |  |
| Short-stay |  |  |  |  |
| CatBoost | 0.75 (0.72, 0.78) | - | 0.68 (0.62, 0.73) | - |
| XGBoost | 0.75 (0.73, 0.78) | 0.40 | 0.66 (0.61, 0.72) | 0.16 |
| RF | 0.75 (0.72, 0.78) | 0.68 | 0.67 (0.62, 0.72) | 0.59 |
| NN | 0.74 (0.71, 0.77) | 0.14 | 0.67 (0.61, 0.72) | 0.51 |
| SVM | 0.74 (0.71, 0.77) | 0.16 | 0.68 (0.63, 0.73) | 0.88 |
| Logistic | 0.74 (0.71, 0.77) | 0.28 | 0.68 (0.63, 0.73) | 0.85 |
| Long-stay |  |  |  |  |
| CatBoost | 0.78 (0.75, 0.81) | - | 0.74 (0.70, 0.79) | - |
| XGBoost | 0.78 (0.75, 0.81) | 0.52 | 0.74 (0.69, 0.78) | 0.45 |
| RF | 0.78 (0.76, 0.81) | 0.51 | 0.74 (0.69, 0.78) | 0.41 |
| NN | 0.77 (0.74, 0.80) | 0.29 | 0.74 (0.69, 0.78) | 0.54 |
| SVM | 0.78 (0.75, 0.81) | 0.97 | 0.73 (0.69, 0.78) | 0.32 |
| Logistic | 0.79 (0.76, 0.81) | 0.28 | 0.74 (0.69, 0.78) | 0.53 |
| Breast cancer |  |  |  |  |
| CatBoost | 0.75 (0.69, 0.82) | - | 0.71 (0.52, 0.90) | - |
| XGBoost | 0.75 (0.68, 0.82) | 0.96 | 0.73 (0.56, 0.90) | 0.62 |
| RF | 0.75 (0.68, 0.82) | 0.99 | 0.75 (0.58, 0.92) | 0.46 |
| NN | 0.74 (0.66, 0.81) | 0.50 | 0.75 (0.60, 0.90) | 0.31 |
| SVM | 0.74 (0.68, 0.81) | 0.56 | 0.76 (0.60, 0.91) | 0.25 |
| Logistic | 0.74 (0.67, 0.81) | 0.52 | 0.76 (0.60, 0.91) | 0.21 |
| Colorectal cancer |  |  |  |  |
| CatBoost | 0.77 (0.74, 0.80) | - | 0.77 (0.71, 0.83) | - |
| XGBoost | 0.77 (0.73, 0.80) | 0.40 | 0.76 (0.70, 0.82) | 0.33 |
| RF | 0.77 (0.74, 0.80) | 0.86 | 0.76 (0.70, 0.82) | 0.32 |
| NN | 0.76 (0.73, 0.80) | 0.15 | 0.78 (0.71, 0.84) | 0.62 |
| SVM | 0.77 (0.73, 0.80) | 0.65 | 0.78 (0.72, 0.84) | 0.36 |
| Logistic | 0.77 (0.74, 0.81) | 0.97 | 0.77 (0.71, 0.83) | 0.93 |
| Liver cancer |  |  |  |  |
| CatBoost | 0.74 (0.67, 0.81) | - | 0.69 (0.59, 0.79) | - |
| XGBoost | 0.73 (0.66, 0.80) | 0.87 | 0.65 (0.55, 0.76) | 0.23 |
| RF | 0.71 (0.64, 0.79) | 0.14 | 0.67 (0.57, 0.77) | 0.52 |
| NN | 0.71 (0.64, 0.79) | 0.26 | 0.66 (0.55, 0.76) | 0.40 |
| SVM | 0.73 (0.64, 0.81) | 0.55 | 0.70 (0.61, 0.79) | 0.80 |
| Logistic | 0.74 (0.66, 0.82) | 0.96 | 0.68 (0.58, 0.78) | 0.81 |
| Lung cancer |  |  |  |  |
| CatBoost | 0.68 (0.63, 0.73) | - | 0.68 (0.63, 0.73) | - |
| XGBoost | 0.68 (0.63, 0.74) | 0.63 | 0.66 (0.60, 0.71) | 0.07 |
| RF | 0.69 (0.63, 0.74) | 0.60 | 0.68 (0.63, 0.73) | 0.91 |
| NN | 0.67 (0.62, 0.72) | 0.63 | 0.65 (0.60, 0.71) | 0.17 |
| SVM | 0.68 (0.62, 0.73) | 0.83 | 0.70 (0.65, 0.75) | 0.34 |
| Logistic | 0.68 (0.63, 0.73) | 0.81 | 0.69 (0.64, 0.74) | 0.54 |
| Pancreas cancer |  |  |  |  |
| CatBoost | 0.69 (0.58, 0.79) | - | 0.62 (0.45, 0.79) | - |
| XGBoost | 0.68 (0.58, 0.78) | 0.72 | 0.60 (0.45, 0.76) | 0.73 |
| RF | 0.72 (0.62, 0.82) | 0.17 | 0.42 (0.25, 0.59) | 0.25 |
| NN | 0.69 (0.58, 0.79) | 0.98 | 0.60 (0.42, 0.79) | 0.70 |
| SVM | 0.72 (0.61, 0.82) | 0.14 | 0.59 (0.39, 0.80) | 0.58 |
| Logistic | 0.72 (0.61, 0.82) | 0.14 | 0.62 (0.43, 0.81) | 0.98 |
| Prostate cancer |  |  |  |  |
| CatBoost | 0.53 (0.40, 0.66) | - | 0.46 (0.28, 0.63) | - |
| XGBoost | 0.47 (0.34, 0.60) | 0.65 | 0.51 (0.33, 0.69) | < 0.001 |
| RF | 0.57 (0.45, 0.68) | 0.33 | 0.55 (0.32, 0.79) | 0.65 |
| NN | 0.48 (0.34, 0.62) | 0.39 | 0.54 (0.31, 0.76) | 0.70 |
| SVM | 0.58 (0.47, 0.70) | 0.22 | 0.67 (0.46, 0.89) | 0.29 |
| Logistic | 0.57 (0.46, 0.68) | 0.30 | 0.64 (0.36, 0.92) | 0.44 |
| Stomach cancer |  |  |  |  |
| CatBoost | 0.80 (0.76, 0.84) | - | 0.81 (0.69, 0.92) | - |
| XGBoost | 0.80 (0.76, 0.84) | 0.61 | 0.77 (0.65, 0.90) | 0.03 |
| RF | 0.80 (0.76, 0.84) | 0.85 | 0.79 (0.66, 0.91) | 0.23 |
| NN | 0.80 (0.76, 0.84) | 0.63 | 0.78 (0.66, 0.90) | 0.16 |
| SVM | 0.80 (0.76, 0.84) | 0.91 | 0.78 (0.66, 0.90) | 0.06 |
| Logistic | 0.81 (0.77, 0.85) | 0.64 | 0.79 (0.67, 0.91) | 0.36 |
| Stage 0-I |  |  |  |  |
| CatBoost | 0.77 (0.74, 0.81) | - | 0.68 (0.62, 0.74) | - |
| XGBoost | 0.78 (0.74, 0.81) | 0.70 | 0.67 (0.62, 0.73) | 0.76 |
| RF | 0.77 (0.74, 0.81) | 0.55 | 0.67 (0.61, 0.73) | 0.76 |
| NN | 0.76 (0.72, 0.79) | 0.02 | 0.67 (0.60, 0.73) | 0.58 |
| SVM | 0.76 (0.73, 0.80) | 0.09 | 0.66 (0.60, 0.73) | 0.41 |
| Logistic | 0.76 (0.73, 0.80) | 0.10 | 0.67 (0.61, 0.73) | 0.48 |
| Stage II |  |  |  |  |
| CatBoost | 0.77 (0.73, 0.81) | - | 0.72 (0.67, 0.78) | - |
| XGBoost | 0.77 (0.73, 0.80) | 0.83 | 0.70 (0.65, 0.76) | 0.06 |
| RF | 0.78 (0.74, 0.82) | 0.05 | 0.71 (0.65, 0.76) | 0.08 |
| NN | 0.77 (0.73, 0.81) | 0.63 | 0.69 (0.63, 0.75) | 0.07 |
| SVM | 0.77 (0.74, 0.81) | 0.52 | 0.71 (0.65, 0.77) | 0.36 |
| Logistic | 0.78 (0.74, 0.81) | 0.07 | 0.71 (0.66, 0.77) | 0.39 |
| Stage III |  |  |  |  |
| CatBoost | 0.75 (0.71, 0.80) | - | 0.73 (0.63, 0.82) | - |
| XGBoost | 0.76 (0.72, 0.80) | 0.55 | 0.71 (0.62, 0.81) | 0.34 |
| RF | 0.75 (0.70, 0.79) | 0.52 | 0.72 (0.63, 0.81) | 0.79 |
| NN | 0.75 (0.71, 0.80) | 0.94 | 0.72 (0.63, 0.81) | 0.76 |
| SVM | 0.76 (0.72, 0.80) | 0.44 | 0.75 (0.66, 0.83) | 0.12 |
| Logistic | 0.76 (0.72, 0.80) | 0.41 | 0.75 (0.67, 0.84) | 0.05 |
| Stage IV |  |  |  |  |
| CatBoost | 0.75 (0.69, 0.81) | - | 0.78 (0.70, 0.86) | - |
| XGBoost | 0.75 (0.68, 0.81) | 0.85 | 0.79 (0.71, 0.87) | 0.55 |
| RF | 0.74 (0.67, 0.80) | 0.45 | 0.80 (0.72, 0.87) | 0.32 |
| NN | 0.73 (0.66, 0.80) | 0.17 | 0.81 (0.74, 0.88) | 0.25 |
| SVM | 0.72 (0.66, 0.79) | 0.08 | 0.80 (0.73, 0.88) | 0.18 |
| Logistic | 0.73 (0.66, 0.80) | 0.18 | 0.80 (0.72, 0.87) | 0.39 |

AUC = the area under the receiver operating characteristic curve; CatBoost = category boosting; Logistic = logistic regression; NN = neural networks; RF = random forest; SVM = support vector machine; XGBoost = extreme gradient boosting.


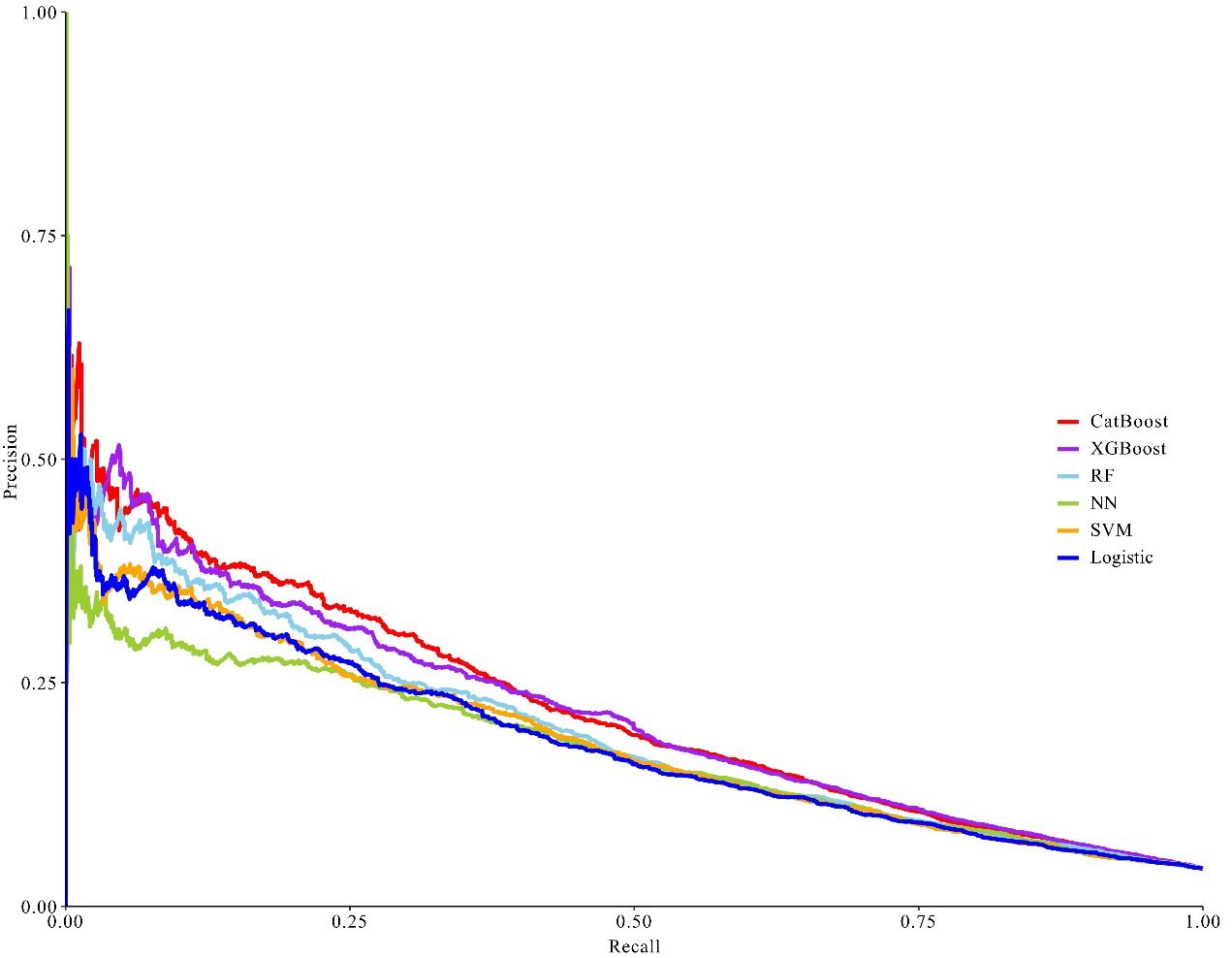


Figure S1. Precision-recall curve of six machine learning models in training set

CatBoost = category boosting; Logistic = logistic regression; NN = neural networks; RF = random forest; SVM = support vector machine; XGBoost = extreme gradient boosting.
